# The top 100 most cited articles on pediatric respiratory syncytial virus pneumonia over the last 30 years: a bibliometric analysis

**DOI:** 10.1186/s41182-025-00862-x

**Published:** 2025-11-27

**Authors:** Fei Luo, Chanchan Hu, Qian Liu, Naixu Liu, Kang Lian, Demei Wu, Zijian Shao, Yuanyuan Wang, Mingchen Jiang, Bin Yuan

**Affiliations:** 1 Department of Pediatrics, Affiliated Hospital of Nanjing University of Chinese Medicine, Nanjing, 210029 China; 2 Department of Pediatrics, Suqian Affiliated Hospital of Nanjing University of Chinese Medicine, Suqian, 223800 China

**Keywords:** Respiratory syncytial virus pneumonia, Pediatric, Bibliometric analysis, Visualization, Web of science

## Abstract

**Background:**

Respiratory syncytial virus (RSV) is a leading cause of acute lower respiratory infections in humans, contributing to a substantial burden on both families and society. To date, no bibliometric studies have specifically addressed RSV pneumonia. We therefore employed bibliometric methods to analyze the top 100 most-cited articles in this field, aiming to construct a visual knowledge map and quantitatively identify current research hotspots and emerging trends.

**Methods:**

We retrieved relevant publications from the Web of Science Core Collection (WoSCC) database. Using Microsoft Excel 2019, CiteSpace 6.2.R4, and VOSviewer 1.6.18, we performed a visual analysis of annual publication trends, countries, institutions, authors, journals, and keywords.

**Results:**

The 100 most-cited articles received a total of 15,949 citations, with individual citation counts ranging from 46 to 2846 and a median of 74. The United States contributed the most publications, and the Centers for Disease Control and Prevention (CDC) was the most productive institution. The most prolific authors were Cohen, Cheryl; Graham, Barney S; Anderson, LJ; and Ramilo, O. The Pediatric Infectious Disease Journal published and received the most citations in this domain. “Bronchiolitis” was identified as the keyword with the strongest citation burst.

**Conclusion:**

Current research on RSV pneumonia remains focused on pathogenesis, treatment, and prognosis. The development of new antiviral drugs and immunoprophylaxis strategies continues to be a central direction for future studies.

## Introduction

Respiratory syncytial virus (RSV) is a major pathogen responsible for acute lower respiratory tract infections, hospitalizations, and deaths in humans [1]. Initially isolated from chimpanzees in 1956, it is named for its characteristic induction of cell fusion and syncytia formation in cultured cells. Based on host specificity, RSV is classified into human respiratory syncytial virus (HRSV, first isolated from an infant in 1957), bovine respiratory syncytial virus (BRSV), and mouse respiratory syncytial virus (MRSV). Virologically, RSV was formerly placed within the genus Pneumovirus of the family Paramyxoviridae. However, the International Committee on Taxonomy of Viruses (ICTV) reclassified it into the genus Orthopneumovirus within the family Pneumoviridae in 2015, and in 2016, HRSV was formally renamed Human Orthopneumovirus [2]. RSV primarily infects infants, the elderly, and immunocompromised individuals, with an estimated 33 million new global cases annually [3], resulting in 118,000 deaths each year among children under five [4]. The burden is especially high in low-and middle-income countries. Furthermore, RSV is a significant risk factor for asthma, recurrent wheezing, and impaired lung function. In children under 3 years of age, the incidence of recurrent wheezing after RSV infection ranges from 4 to 47%, while that of asthma ranges from 8 to 76% [5]. Impaired lung function is associated with an increased risk of cardiovascular disease and premature mortality in adulthood [6]. Clinical symptoms include nasal congestion, rhinorrhea, cough, dyspnea, and fever, with severe cases progressing to respiratory failure [7]. Current preventive measures rely on monoclonal antibodies such as Palivizumab and Nirsevimab, which target the viral fusion (F) protein to block host cell entry, substantially reducing hospitalization rates in high-risk pediatric populations.

Bibliometrics provides a quantitative approach to reveal disciplinary trends and frontiers by analyzing the distribution and relationships among knowledge carriers [8]. Commonly used tools include CiteSpace [9], which visualizes knowledge structures and detects emerging themes, and VOSviewer [10], which is particularly suited for co-citation network analysis. Among citation databases, Web of Science (WOS) is the most authoritative and covers the broadest range of disciplines, with an emphasis on natural sciences [11]. Although Scopus includes a wide selection of journals, it suffers from delayed indexing; Google Scholar often provides incomplete citation data, and PubMed lacks a native citation analysis module [12].

To date, no bibliometric study has specifically addressed RSV pneumonia. This work presents the first bibliometric analysis of the top 100 most-cited articles on RSV pneumonia from the Web of Science Core Collection (WoSCC). Using methods such as co-citation clustering, keyword emergence analysis, and knowledge mapping, we systematically examine the evolution, core topics, and research frontiers in this field. Our objective is to offer a quantitative foundation and conceptual framework to inform future investigative priorities.

## Materials and methods

### Data selection

**Fig. 1:**
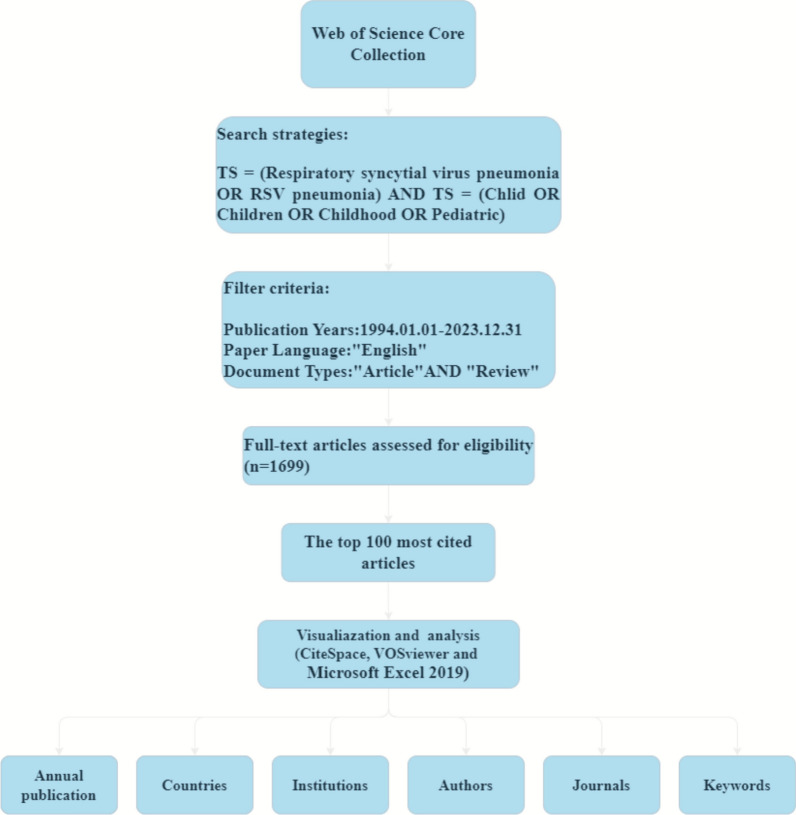
Flowchart of literature search. TS stands for Topic Search

On December 24, 2024, we searched the WoSCC for RSV pneumonia-related literature, ranking the results by citation count in descending order and selecting the 100 most highly cited articles. Our search criteria included the terms “respiratory syncytial virus pneumonia” or “RSV pneumonia” in combination with “Child,” “Children,” “Childhood,” or “Pediatric.” The study focused on English-language articles and reviews, excluding abstracts, editorials, conference papers, book chapters, and retracted publications. The search encompassed a 30-year period from January 1, 1994, to December 31, 2023, with the results obtained promptly on the same day. Study selection and data extraction were performed independently by two authors. Any discrepancies were resolved by discussion or by consulting a third author to reach a consensus. Figure 1 illustrates the literature search process.

### Data analysis

Extract key information from the eligible articles, such as title, authors, countries, institutions, keywords, and publication year. The collected data was then processed utilizing Microsoft Excel 2019, along with CiteSpace 6.1.R6 and VOSviewer 1.6.18 software.

### Quality control

To ensure methodological rigor and reporting transparency, this study adhered strictly to the Preliminary Guideline for Reporting Bibliometric Reviews of the Biomedical Literature (BIBLIO) [13]. This checklist offers a comprehensive framework for bibliometric research, and its recommendations guided all stages of data collection, processing, analysis, and interpretation.

## Result

### Characteristics of the included articles

This study systematically searched the WoSCC database for literature related to RSV pneumonia in children from 1994 to 2023, yielding a total of 1699 research records. Through descending citation frequency, the 100 most influential articles were selected for in-depth analysis, including 86 research papers and 14 review articles.

**Fig. 2:**
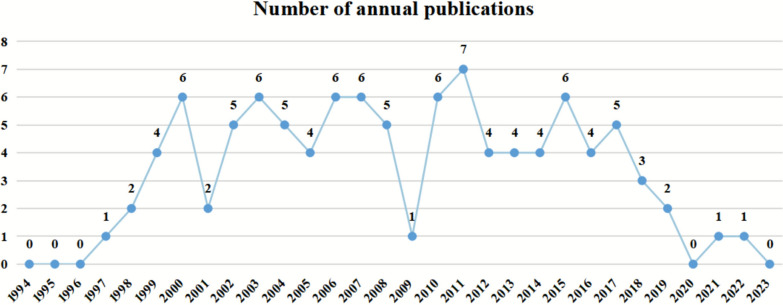
Publishing years of the 100 top-cited articles on RSV pneumonia

Table 1 lists the top 10 most-cited articles in the field of pediatric RSV pneumonia research. Figure 2 illustrates the annual distribution of the top 100 most-cited articles from 1994 to 2023, where the x-axis represents the year, and the y-axis corresponds to the annual publication volume. Analysis revealed that these 100 highly cited articles have been cited a total of 15,949 times, with individual articles being cited between 46 and 2846 times, and a median citation count of 74. Regarding annual distribution, the publication volume fluctuated between 1 and 7 articles, peaking in 2011 (n = 7). Notably, only 2 articles were cited over 2000 times, and 3 articles were cited over 1000 times.

**Table 1:** Top 10 most-cited articles on RSV pneumonia

Rank	Article title	Journal	Publication year	IF	Total citation	Average Annual Citation
1	Mortality associated with influenza and respiratory syncytial virus in the United States	Jama-Journal Of The American Medical Association	2003	63.1	2864	130.18
2	Global burden of acute lower respiratory infections due to respiratory syncytial virus in young children: a systematic review and meta-analysis	Lancet	2010	98.4	2059	137.27
3	Global, regional, and national disease burden estimates of acute lower respiratory infections due to respiratory syncytial virus in young children in 2015: a systematic review and modelling study	Lancet	2017	98.4	1513	189.13
4	Bronchiolitis-associated mortality and estimates of respiratory syncytial virus-associated deaths among US children, 1979–1997	Journal Of Infectious Diseases	2001	5	341	14.21
5	A role for immune complexes in enhanced respiratory syncytial virus disease	Journal Of Experimental Medicine	2002	12.6	297	12.91
6	Development of motavizumab, an ultra-potent antibody for the prevention of respiratory syncytial virus infection in the upper and lower respiratory tract	Journal Of Molecular Biology	2007	4.7	289	16.06
7	Viral Load Drives Disease in Humans Experimentally Infected with Respiratory Syncytial Virus	American Journal Of Respiratory And Critical Care Medicine	2010	19.3	243	16.2
8	Structural characterization of the human respiratory syncytial virus fusion protein core	Proceedings Of The National Academy Of Sciences Of The United States Of America	2000	9.4	239	9.56
9	Whole Blood Gene Expression Profiles to Assess Pathogenesis and Disease Severity in Infants with Respiratory Syncytial Virus Infection	Plos Medicine	2013	10.5	229	19.08
10	Respiratory Syncytial Virus Activates Innate Immunity through Toll-Like Receptor 2	Journal Of Virology	2009	4.0	215	13.44

This study involved 643 researchers from 42 countries, spanning 243 research institutions, and the research outcomes were published in 60 academic journals, demonstrating a broad trend of international collaboration and exchange.

### Analysis of countries

**Fig. 3:**
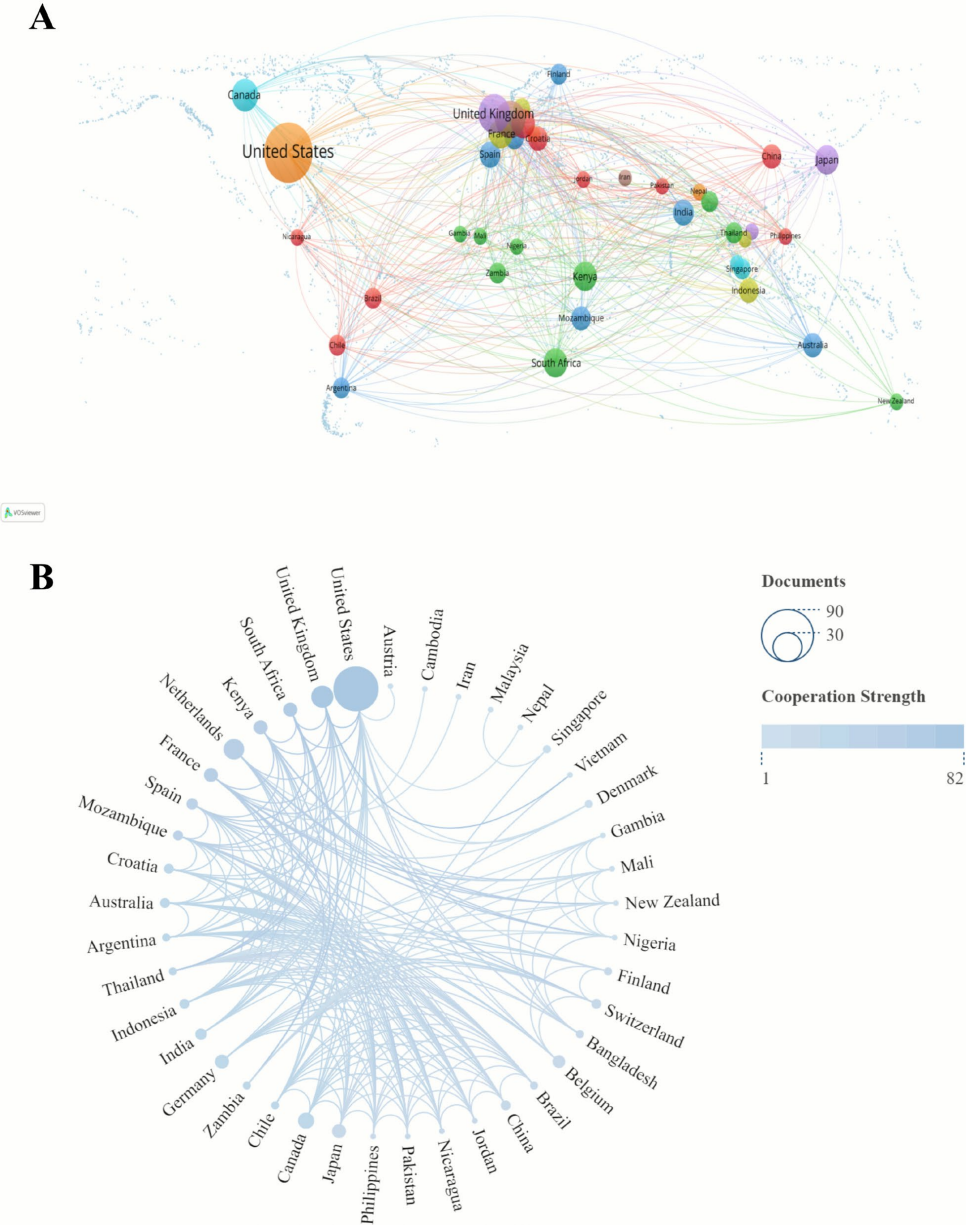
Analysis of the countries. **A** World the cooperation intensity map. **B** A circle dia-gram that evaluates international collaboration between clusters

As shown in Table 2 and Fig. 3, a total of 41 countries worldwide have participated in highly cited research in the field of RSV pneumonia in children. Analyzing the number of publications by country, the United States ranks first with 63 articles, followed by the United Kingdom (16 articles) and the Netherlands (14 articles) in second and third places, respectively. In terms of total citation frequency by country, the United States (11,631 citations), the United Kingdom (3710 citations), and Kenya (2699 citations) form the core knowledge production group. Notably, in terms of average citations per article, Kenya (386 citations/article), Japan (373 citations/article), and France (356 citations/article) demonstrate significant academic influence.

**Table 2:** Top 10 countries of top 100 most-cited articles on RSV pneumonia

Rank	Countries	Publications	Citations	Mean citations per article
1	United States	63	11,631	185
2	United Kingdom	16	3710	232
3	Netherlands	14	1160	83
4	Canada	8	667	83
5	France	7	2495	356
6	South Africa	7	639	91
7	Germany	7	443	63
8	Japan	7	2608	373
9	Kenya	7	2699	386
10	Belgium	5	537	107

Figure 3, as an analysis diagram of the national cooperation network for RSV pneumonia, provides a visual representation of the global research landscape. Figure 3a clearly illustrates the geographical distribution of RSV pneumonia publications by country. Evidently, the primary research countries for RSV pneumonia are concentrated in North America and Europe. Leveraging their advanced scientific research facilities, substantial research funding, and top-tier scientific talent teams, countries in these two regions have achieved remarkable research outcomes in this field, leading global RSV pneumonia research. Figure 3b further reflects international cooperation among clusters. The size of the nodes in the figure intuitively represents the number of publications from each country; a larger node indicates more significant achievements in RSV pneumonia research by that country. The connecting lines clearly indicate the strength of cooperation between countries; thicker lines signify closer scientific collaboration and richer cooperation outcomes between the two countries.

### Analysis of institutions

**Fig. 4:**
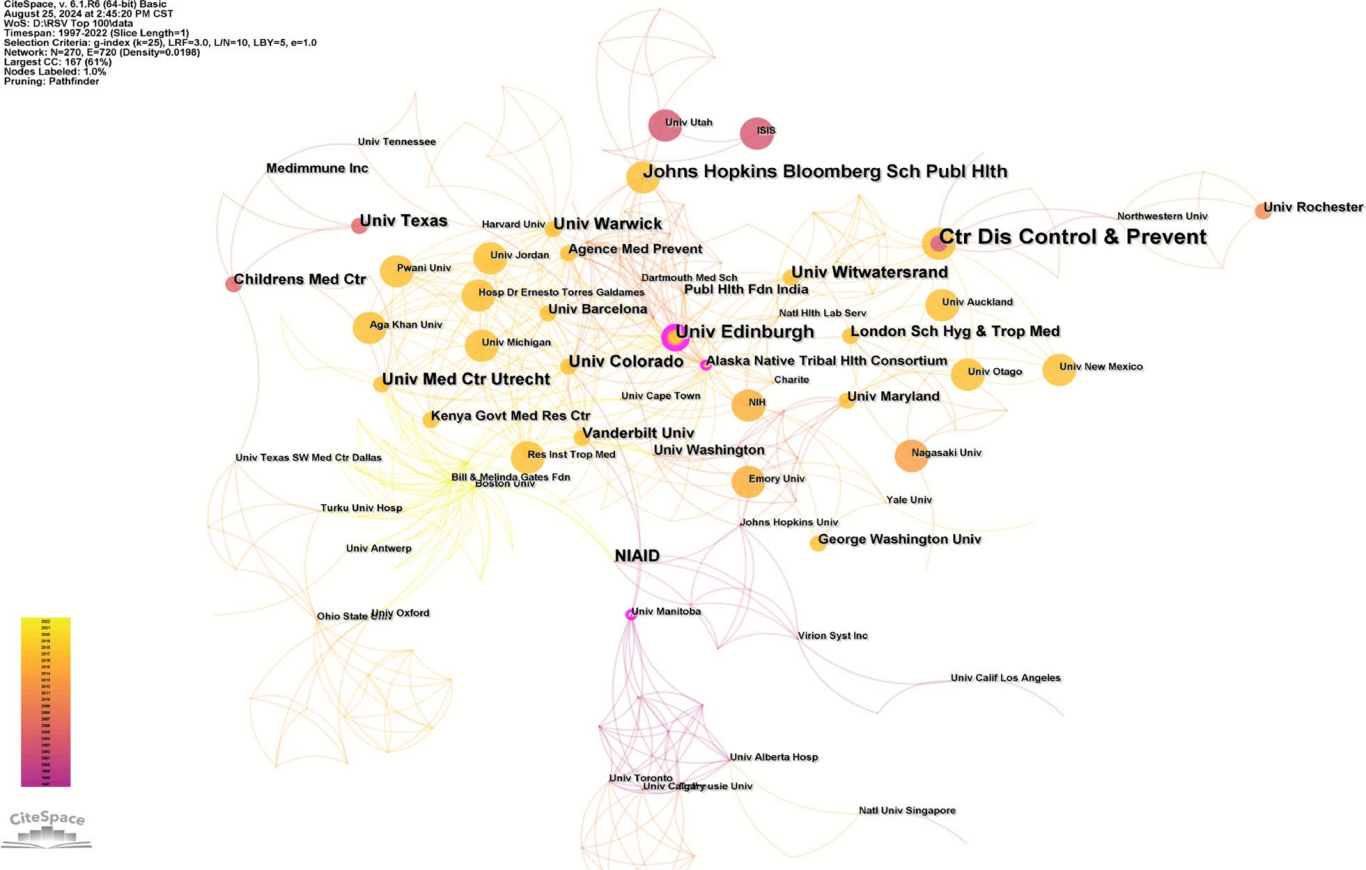
Institutions of the top 100 most-cited articles on RSV pneumonia

According to Table 3 and Fig. 4, 243 research institutions worldwide have participated in the knowledge production of highly cited studies in the field of pediatric RSV pneumonia. The top two institutions with the most published papers are the Centers for Disease Control and Prevention (CDC) in the United States (9 papers) and the University of Edinburgh (6 papers). In terms of citation counts, the top two institutions are the CDC (3776 citations) and the University of the Witwatersrand (2371 citations) (Table 3).

**Table 3:** Top 10 institutions of the top 100 most-cited articles on RSV pneumonia

Rank	Institutions	Publications	Citations	Centrality	Country
1	Centers for Disease Control and Prevention	9	3776	0.09	USA
2	University of Edinburgh	6	2301	0.16	UK
3	Johns Hopkins Bloomberg School of Public Health	6	2230	0.04	USA
4	University of Warwick	5	2313	0.09	UK
5	University of Colorado	5	2213	0.08	USA
6	University Medical Center Utrecht	5	116	0.06	Netherlands
7	University of the Witwatersrand	5	2371	0.05	South Africa
8	National Institute of Allergy and Infectious Diseases	5	510	0.04	USA
9	University of Texas	5	557	0.02	USA
10	London School of Hygiene and Tropical Medicine	4	220	0.06	UK

Betweenness centrality is an indicator that measures the importance of a node in a network. A higher centrality value indicates that the node plays a bridging role in communicating with other nodes [14]. Nodes with a centrality value > 0.1 are considered relatively important. Empirical analysis shows that the University of Edinburgh (centrality = 0.16), as the only node breaking the threshold (> 0.1) (marked in purple), plays a key bridging function in cross-regional scientific research collaboration. Additionally, despite having both a high number of publications and citations, the CDC’s centrality value (0.09) did not reach the threshold, suggesting that its research relies more on internal resources rather than international collaboration.

### Analysis of authors

**Fig. 5:**
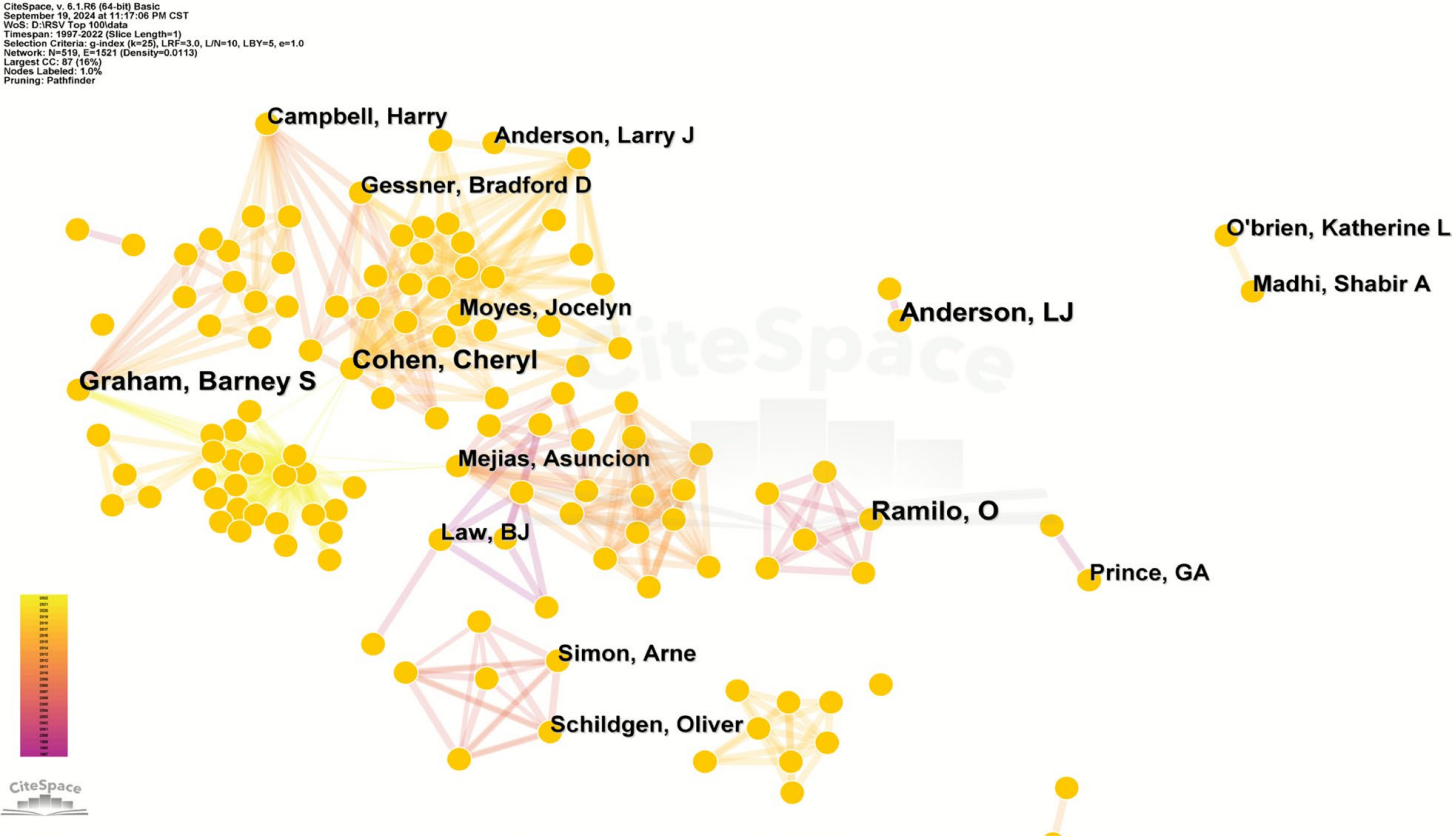
Authors of the top 100 most-cited articles on RSV pneumonia

The highly cited literature in the field of pediatric RSV pneumonia involves 519 researchers from around the globe. Table 4 showcases the top 10 authors who have contributed the most to these 100 articles. Cohen, Cheryl, Graham, Barney S, Anderson, LJ, and Ramilo, O are tied for the first place as the most prolific authors, each with 4 articles published. Graham, Barney S, Anderson, LJ, and Ramilo, O are all from the United States, while Cohen, Cheryl is from South Africa. As illustrated in Fig. 5, the author collaboration network exhibits a multi-center discrete distribution. Despite the presence of cross-institutional collaborations, none of the nodes’ betweenness centrality exceeded the significance threshold of 0.1, indicating no high-centrality authors were observed.

**Table 4:** Top 10 authors of top 100 most-cited articles on RSV pneumonia

Rank	Author	Affiliations	Publications	Country
1	Cohen, Cheryl	University of the Witwatersrand	4	South Africa
2	Graham, Barney S	National Institute of Allergy and Infectious Diseases	4	USA
3	Anderson, LJ	Centers for Disease Control and Prevention	4	USA
4	Ramilo, O	University of Texas	4	USA
5	Mejias, Asuncion	Ohio State University College of Medicine	3	USA
6	Campbell, Harry	University of Edinburgh	3	UK
7	Gessner, Bradford D	Agence de Médecine Préventive	3	France
8	Prince, GA	Virion Systems, Rockville, Maryland	3	USA
9	Simon, Arne	University of Bonn	3	Germany
10	Madhi, Shabir A	University of the Witwatersrand	3	South Africa

### Analysis of Journals

**Fig. 6:**
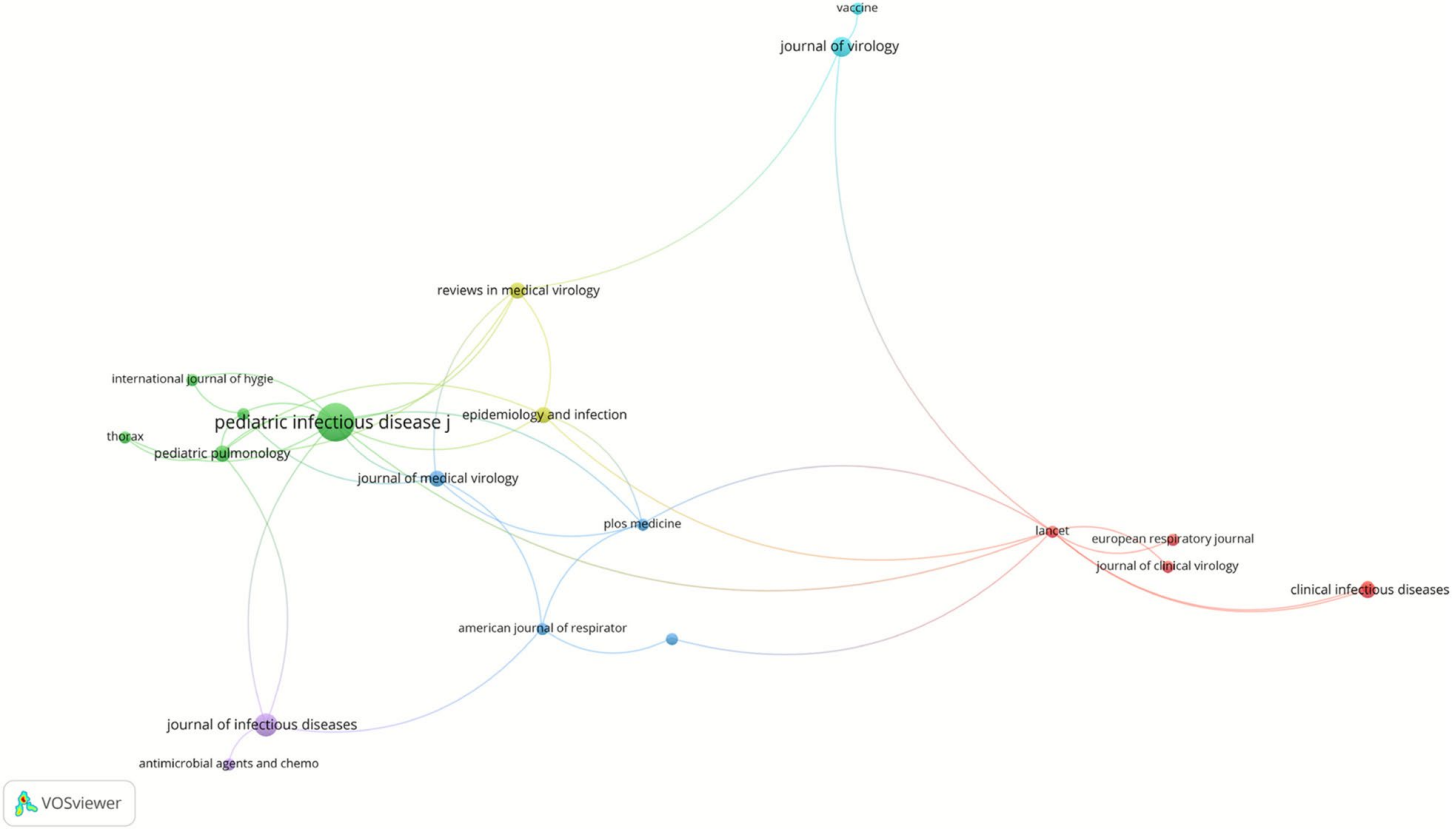
Journals of the top 100 most-cited articles on RSV pneumonia

Based on Fig. 6 and Table 5, the top 100 highly cited articles in the field of pediatric RSV pneumonia were published across 60 academic journals. The *Pediatric Infectious Disease Journal* published the highest number of articles (n = 10), followed by the Journal of Infectious Diseases (n = 5) and the Journal of Virology (n = 4). Among the top 10 journals, 5 belong to the JCR Q1 quartile, 3 belong to the Q2 quartile, and 2 belong to the Q3 quartile.

**Table 5:** Top 10 journals of top 100 most-cited articles on RSV pneumonia

Rank	Journal	Publications	IF (2023)	JCR (2023)
1	Pediatric Infectious Disease Journal	10	2.89	Q3
2	Journal of Infectious Diseases	5	5	Q2
3	Journal of Virology	4	4	Q2
4	Clinical Infectious Diseases	3	8.19	Q1
5	Epidemiology and Infection	3	2.5	Q3
6	Journal of Medical Virology	3	6.79	Q1
7	Pediatric Pulmonology	3	2.7	Q1
8	Reviews in Medical Virology	3	9	Q1
9	American Journal of Respiratory and Critical Care Medicine	2	19.3	Q1
10	Antimicrobial Agents and Chemotherapy	2	4.09	Q2

### Analysis of keywords

The top 100 most-cited articles in this study contained 453 keywords. Table 6 lists the top 15 high-frequency keywords, with “respiratory syncytial virus” (appearing 48 times), “Child” (appearing 42 times), “infant” (appearing 32 times), and “infection” (appearing 31 times) being the keywords that appeared more than 30 times.

**Table 6:** Top 15 keywords of top 100 most-cited articles on RSV pneumonia

Rank	Keyword	Occurrences	Centrality
1	Respiratory syncytial virus	48	0.10
2	Child	42	0.36
3	Infant	32	0.23
4	Infection	31	0.38
5	Disease	24	0.14
6	Risk	23	0.07
7	Bronchiolitis	23	0.11
8	Pneumonia	22	0.03
9	Epidemiology	17	0.12
10	Mouse	12	0.13
11	Tract infection	12	0
12	Influenza	10	0.04
13	Immune prophylaxis	10	0.10
14	Hospitalization	9	0.04
15	Human metapneumovirus	9	0.10

**Fig. 7:**
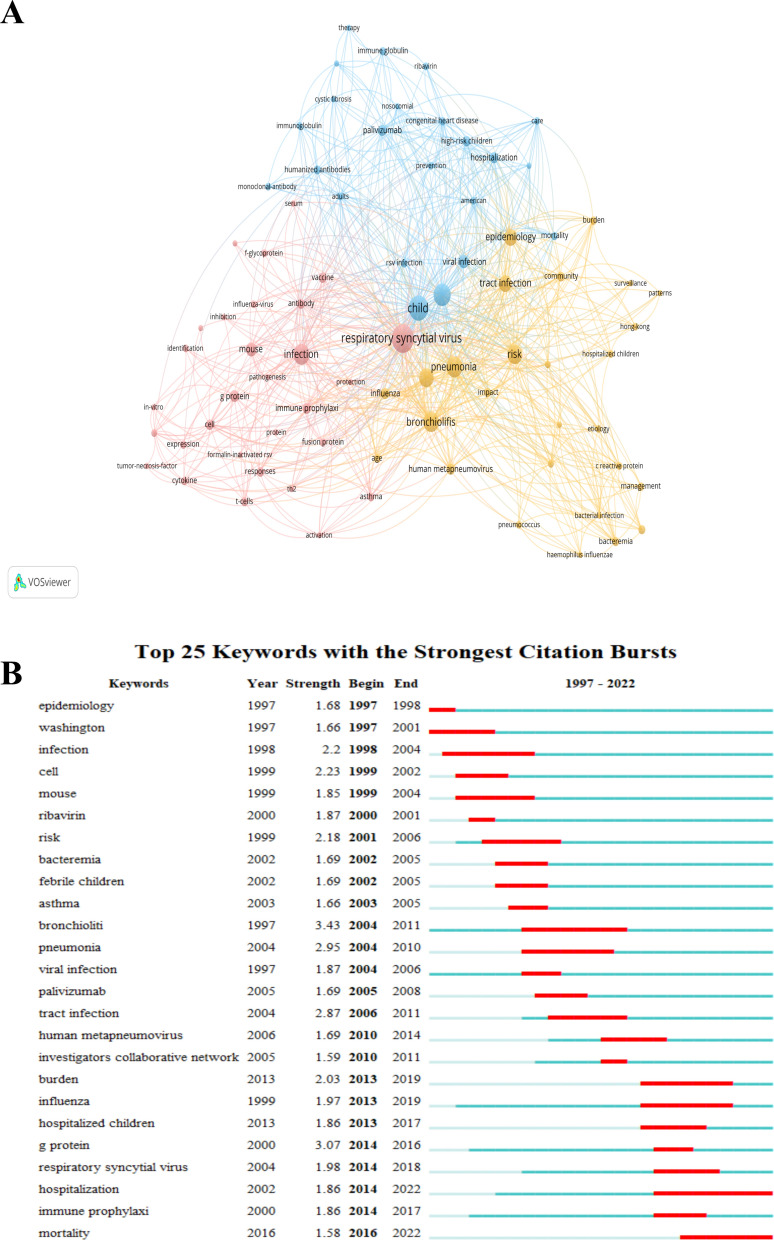
Keywords of the top 100 most-cited articles on RSV pneumonia. **A** Visualization of keywords. **B** The top 25 keywords with the strongest citation bursts

From the keyword co-occurrence graph in Fig. 7a, it can be seen that the red phrase group involves RSV infection and immune response, such as “activation”, “immune prophylaxis”, “cytokine”, “T-cells”, “viral infection”, etc. These keywords indicate that this cluster mainly focuses on the immune response triggered by RSV infection, cytokine release, and immune prophylaxis mechanisms, especially the body’s immune response after RSV infection and the virus’s immune evasion mechanisms. The yellow phrase group includes risks and pathologies related to pneumonia and bacterial infections, such as “bacteremia”, “bacterial infection”, “community-acquired pneumonia”, “risk”, “epidemiology”, “burden”, etc. These keywords show that this cluster mainly concerns the pathophysiology of pneumonia caused by bacterial infections, community-acquired pneumonia, pathological burden, and epidemiological data, with an emphasis on co-infections of RSV and bacteria, as well as complications such as pneumonia. The blue phrase group covers the clinical manifestations and treatment of RSV infection in children, including “child”, “infants”, “high-risk children”, “hospitalization”, “therapy”, “prevention”, “monoclonal-antibody”, etc. These keywords suggest that this cluster primarily focuses on the clinical features, treatment strategies, and preventive measures of RSV infection in children, particularly the incidence of RSV in high-risk children, hospitalization situations, and the effectiveness of monoclonal antibody therapy.

Figure 7b illustrates the top 25 keywords based on burst analysis intensity. The top five keywords with the highest burst intensities are “bronchiolitis” (3.43), “g protein” (3.07), “pneumonia” (2.95), “tract infection” (2.87), and “cell” (2.23). The earliest emerging keywords are “Washington” and “epidemiology”, while the most recent emerging keyword is “hospitalization”.

## Discussion

Employing bibliometric methods, this study identified and rigorously analyzed 100 highly influential articles in the field of RSV pneumonia. By systematically examining research trends, core themes, and emerging frontiers, it integrates foundational studies with recent advances. The resulting academic landscape reveals the evolution of knowledge in this domain, offering both quantitative evidence and theoretical support for understanding the developmental trajectory of RSV pneumonia research.

### General information

Our research results indicate that the median citation count for the top 100 articles on RSV pneumonia is 74. In terms of document type, over 86% of the top 100 cited papers are original articles. The peak number of the top 100 cited articles occurred in 2011, with 7 articles.

In terms of countries and institutions, the United States holds a dominant position in the field of RSV pneumonia research, followed closely by the United Kingdom. From a national geographical perspective, these two countries occupy a central role in this research area. The top 10 institutions are mostly located in North America and Europe, indicating that the primary research activities on RSV pneumonia are concentrated in these regions. The Centers for Disease Control and Prevention (CDC) and the University of Edinburgh stand out as the two most productive institutions, playing a pivotal role in RSV pneumonia research. As a leading institution in the field of RSV research in Europe, the University of Edinburgh has systematically established a European RSV epidemiological database through leading and implementing research projects such as RESCEU [15] (Respiratory Syncytial Virus Consortium in Europe) and PROMISE [16] (Preventing Severe Respiratory Syncytial Virus Infections in Infants). These projects not only provide key evidence-based support for the European Union to develop RSV monitoring guidelines, but also directly promote the scientific decision-making process of incorporating monoclonal antibody prevention strategies into the immunization programs of member states. The Centers for Disease Control and Prevention (CDC) in the United States has conducted systematic epidemiological research on Respiratory Syncytial Virus (RSV) infection in adults, building a quantitative database covering hospitalization rates, outpatient visit rates, and the economic burden of the disease. The research findings provide profound evidence-based support for developing intervention strategies for high-risk groups with RSV [17].

In the field of RSV pneumonia research, Professor Cohen, Cheryl focuses on studying key pathogens, including influenza, RSV, SARS-CoV-2, and pneumococcus [18, 19]. Professor Graham, Barney S. from NIAID is a prominent scholar in the RSV field. His innovative work on the RSV prefusion F structure has established a “game-changing” approach for structure-based vaccine design, which is currently being applied to the development of vaccines for coronaviruses and other important pathogens, including Ebola and influenza [20]. Professor Anderson, LJ’s team mainly studies the pathogenic mechanism of RSV to guide vaccine development [21]. Professor Ramilo, O is also dedicated to the development of an RSV vaccine [22].

Regarding journals, the *Pediatric Infectious Disease Journal* is the most prolific publication, holding significant academic importance in the field of RSV pneumonia. Moreover, most of the listed journals fall into the Q1 or Q2 categories, indicating that these journals hold a high academic status in RSV pneumonia research and are widely esteemed by scholars.

### Research hotspots

Combining keyword co-occurrence and cluster analysis, the current research hotspots of Respiratory Syncytial Virus (RSV) pneumonia can be summarized into three aspects: pathophysiology, treatment, and prognosis.

#### Pathophysiology

RSV infection primarily affects the respiratory system, with the main mechanisms being airway obstruction, bronchial smooth muscle spasm, and subsequent airway hyperresponsiveness [23].

Mechanical airway obstruction: RSV invades the epithelial cells of the trachea, bronchioles, and alveoli, triggering the shedding of ciliated epithelial cells. This process creates a vicious cycle: shed cells accumulate in the airways along with inflammatory cells such as neutrophils and lymphocytes, contributing to luminal narrowing due to mucus hypersecretion and mucosal edema. It’s worth noting that neutrophils exacerbate pathological changes through a dual role: ① releasing oxygen free radicals and elastase, which directly damage the epithelial barrier; ② upregulating the expression of TNF-α and IL-13, driving excessive mucus gland secretion.

Neurogenic bronchial spasm: After the epithelial barrier is disrupted, exposed sensory nerve endings release neuropeptides such as substance P, triggering bronchial smooth muscle contraction through the following mechanisms: ① directly activating l-type calcium channels in the ASMC cell membrane, elevating intracellular Ca^2^⁺ concentration; ② stimulating mast cells to release bronchoconstrictor mediators like histamine and leukotrienes; ③ inducing increased cholinergic nerve excitability, promoting acetylcholine release. It’s particularly noteworthy that neurogenic inflammation induced by substance P can produce a continuous amplification effect.

Persistent airway hyperresponsiveness: Airway remodeling after RSV infection involves a complex regulatory network: ① imbalance between β-adrenergic receptor function inhibition and M receptor activation; ② NGF-mediated sensory nerve sensitization; ③ a chronic inflammatory state driven by the Th17/IL-17 axis.

#### Treatment

Current treatment methods include supportive care (such as maintaining electrolyte balance, providing nutrition, and ensuring airway patency), symptomatic treatment (such as antipyretic and analgesic drugs, bronchodilators), antiviral therapy (such as the monoclonal antibody nirsevimab), and immunotherapy (such as monoclonal antibody prophylaxis for high-risk groups) [24]. The use of glucocorticoids and leukotriene receptor antagonists remains controversial.

#### Prognosis

RSV infection is a self-limiting disease, and the vast majority of infected children have a good prognosis without any lasting sequelae. A very small number of children may experience respiratory failure, neurological complications, or even death. In a minority of children, recurrent wheezing and bronchial asthma may occur in the later stages of infection, with some cases resulting in post-infectious bronchiolitis obliterans. Severe RSV infection is associated with an increased risk of recurrent wheezing and asthma in preschool children [24].

Research on RSV is undergoing a major paradigm shift, propelled by innovations in prevention strategies and the influence of the COVID-19 pandemic. The field is gradually moving away from a historically reactive posture, centered on post-infection treatment, toward a new era defined by proactive intervention. This transition is supported by breakthroughs in long-standing research challenges. Recent advances, drawing on structural insights into the pre-fusion F protein, enabled the approval of long-acting monoclonal antibodies like nirsevimab and maternal vaccines in 2023, fundamentally altering the prevention landscape [25, 26]. Investigative priorities have now broadened beyond traditional clinical trials to include four key real-world concerns: real-world effectiveness (RWE), implementation science focused on accessibility and equity, genomic surveillance of viral escape mutations, and mechanisms through which maternal antibodies shape infant immune responses [27–29]. At the same time, the COVID-19 pandemic has substantially reshaped RSV epidemiology, disrupting seasonal trends, introducing the concept of “immunity debt” [30, 31], and stimulating deeper inquiry into the clinical effects of viral co-infection [32]. The success of COVID-19 vaccines, especially mRNA platforms, has also established new technological foundations for RSV vaccine development [33]. In summary, the convergence of preventive advances and pandemic-related changes is driving RSV research into an era of interdisciplinary integration. Future efforts will connect clinical medicine, public health, and viral immunology, as novel and forward-looking scientific questions continue to expand and redefine conventional research priorities.

### Limitation

This study has several limitations. First, methodological biases may have arisen from the inclusion of self-citations, potentially inflating citation counts, and from using citation volume as a screening criterion, which could favor earlier publications, review articles, and studies in high-impact journals while underrepresenting recent original research and specialized journals. Second, limitations pertain to the analytical tools and search strategy: the performance of the bibliometric software depends on data quality and the stability of keyword extraction algorithms, and although the search terms encompassed core pediatric concepts, it is possible that a small number of studies using terms such as “infant” alone were overlooked. Third, systemic biases exist in the data sources due to the restriction to English-language articles within the Web of Science Core Collection, which excludes non-English publications and regional databases and may limit the global representativeness and generalizability of the results.

## Conclusion

This systematic review analyzes the 100 most-cited research articles on pediatric RSV pneumonia from the past three decades, constructing a knowledge map and tracing the evolution of the field. The findings indicate that research on RSV pneumonia centers primarily on pathophysiology, treatment, and prognosis. Developing novel antiviral agents and immunoprophylaxis strategies continues to represent a major direction for future investigation.

## Data Availability

The datasets used and/or analyzed during the current study are available from the corresponding author on reasonable request.
